# Efficacy and tolerability of psychostimulants for symptoms of attention-deficit hyperactivity disorder in preschool children: A systematic review and meta-analysis

**DOI:** 10.1192/j.eurpsy.2023.11

**Published:** 2023-02-15

**Authors:** Hsien-Jane Chiu, Cheuk-Kwan Sun, Yu-Shian Cheng, Ming Yu Wang, Ruu-Fen Tzang, Feng-Li Lin, Ying-Chih Cheng, Weilun Chung

**Affiliations:** 1 Taoyuan Psychiatric Center, Ministry of Health and Welfare, Taoyuan City, Taiwan; 2 Institute of Hospital and Health Care Administration, National Yang Ming Chiao Tung University, Hsinchu, Taiwan; 3 Department of Emergency Medicine, E-Da Hospital, I-Shou University, Kaohsiung, Taiwan; 4 School of Medicine for International Students, College of Medicine, I-Shou University, Kaohsiung, Taiwan; 5 Department of Psychiatry, Tsyr-Huey Mental Hospital, Kaohsiung, Taiwan; 6 Institute of Biomedical Sciences, National Sun Yat-Sen University, Kaohsiung City, Taiwan; 7 Department of Psychiatry, China Medical University Hsinchu Hospital, Hsinchu, Taiwan; 8 Department of Health Services Administration, China Medical University, Taichung City, Taiwan; 9 Department of Psychiatry, Mackay Memorial Hospital, Taipei, Taiwan; 10 Institute of Epidemiology and Preventive Medicine, College of Public Health, National Taiwan University, Taipei City, Taiwan; 11 Research Center of Big Data and Meta-analysis, Wan Fang Hospital, Taipei Medical University, Taipei City, Taiwan

**Keywords:** hyperactivity/impulsivity, inattention, preschool, psychostimulants

## Abstract

**Background:**

There was no previous meta-analysis investigating the efficacy/tolerability of psychostimulants for symptoms of attention-deficit hyperactivity disorder (ADHD) in preschool children.

**Methods:**

Databases including PubMed, the Cochrane Library, EMBASE, ScienceDirect, and ClinicalTrials.gov were searched from inception to March 2022 for randomized controlled trials (RCTs) on therapeutic efficacy of psychostimulants against ADHD symptoms in preschool children (age ≤6 years) compared with placebos. Primary outcomes were (a) changes in ADHD symptoms evaluated by validated rating scales from parents’/teacher’s observation, or (b) post-intervention improvements in neuropsychological performance. Secondary outcomes were risks of adverse events.

**Results:**

Meta-analysis of nine eligible trials including 544 preschool children (mean age=4.86 years, female=11.98%, median treatment duration=4.33 weeks) supported the efficacy of psychostimulants against global symptoms from observations of parents (Hedges’ g=0.6152, p<0.0001) and teachers (Hedges’ g=0.6563, p=0.0039). Efficacy of psychostimulants was also noted against symptoms of inattention and hyperactivity/impulsivity, especially the latter (i.e., main symptoms in preschool children). Moreover, male gender, older age, and longer treatment duration were associated with better efficacy. Regarding adverse events, only the risk of poor appetite was higher in the psychostimulant group (odds ratio [OR]=2.39). However, the qualities of evidence were low to very low, indicating potential discrepancy between the true and estimated effect.

**Conclusions:**

Our results showed that psychostimulants might be beneficial for preschool children with ADHD, especially hyperactivity/impulsivity from teachers’ observation, with tolerable side effects. Nevertheless, the true magnitude of the effect needs to be confirmed with more research due to low to very low certainty of the evidence.

## Introduction

Attention-deficit hyperactivity disorder (ADHD), which is a neurodevelopmental anomaly with an onset in early childhood, affects approximately 5% of school-age children [1] as well as 2.5% of adults worldwide [2]. Despite the high prevalence, the figures may still be underestimated because of the challenge of making an accurate diagnosis for preschool children [3]. Although the core symptoms of ADHD including inattention, hyperactivity, and impulsivity are hallmarks of the disease among school-age children (6–12 years of age) and adults [4], the symptoms are more obscure among preschool children (<6 years of age) who may present with disruptive behavior such as tantrums and aggression as well as psychosocial difficulties (e.g., parent–child conflict) [5].

Both pharmacological (e.g., psychostimulants and non-stimulants) and non-pharmacological treatments (e.g., behavior therapy) against the symptoms of ADHD are commonly recommended clinical strategies for those diagnosed with ADHD [6, 7]. Focusing on pharmacologic regimens, a previous large-scale meta-analysis supported the prescription of methylphenidate (i.e., a psychostimulant) for children diagnosed with ADHD [8]. Psychostimulants including methylphenidate and amphetamine derivatives have also been recommended as the first-line treatment according to clinical guidelines for school-aged children diagnosed with ADHD [9]. Nevertheless, there are concerns about the quality of previous randomized controlled trials (RCT) investigating the efficacy of psychostimulants and also their long-term efficacy [10, 11]. Pharmacological treatment for ADHD in preschool children has posed an even greater challenge for clinicians, given the limited evidence in support of its efficacy and the uncertainties about medication-related side effects in this particular population [12]. A previous study focusing on preschoolers reported greater evidence of effectiveness in parent behavior training compared to methylphenidate for the treatment of preschoolers at risk for ADHD. [3]. Consistently, the American Academy of Pediatrics (AAP) recommends parent training in behavior management rather than the pharmacologic approach as the first line of treatment taking into consideration the advantage and efficacy of parent training, the increased susceptibility of young children to medication-related side effects compared to older children as well as the unclear long-term effects of ADHD medications [12]. As a result, psychostimulants were only considered for those who exhibited unsatisfactory responses to behavioral therapies [9].

Nevertheless, not only are preschool children with symptoms of ADHD equally susceptible to psychosocial impairments and developmental problems compared to school-aged children [13] but they also have a higher risk of future psychiatric comorbidities and lower school readiness than that in their healthy counterparts [14, 15]. Although behavioral therapies were found to be effective and are generally recommended as a first-line intervention [16], behavioral problems remain an issue in a significant portion of children who received only behavioral treatments [3]. Moreover, given that parents of ADHD children are themselves at a higher risk of ADHD [17], the effectiveness of parent training may be compromised [18]. Furthermore, previous studies found that not only were children with ADHD prone to injuries [19, 20], but this risk was also higher in the preschool population [19]. Indeed, a previous survey also showed that more than 50% of clinicians may recommend medication use for preschool children diagnosed with ADHD due to concerns over the impact of impulsiveness on their physical safety. To date, the issue of the efficacy and safety of psychostimulant prescription for ADHD in preschool children remains debated.

Therefore, the aims of this study were to investigate the efficacy of psychostimulants against the core and global symptoms of ADHD, the associated adverse events, and the factors affecting their effectiveness among preschool children.

## Materials and Methods

### Data sources and search strategy

This systematic review and meta-analysis was conducted according to the PRISMA statement guidelines [21]. We searched electronic databases including PubMed, the Cochrane Library, EMBASE, ScienceDirect, and ClinicalTrials.gov from inception to March 2022 for eligible RCTs. The protocol of this study was registered with the PROSPERO systematic review protocol registry (Number CRD42022329966).

The literature search was conducted by three independent researchers (YC Cheng, YS Cheng, and CK Sun) by using mainly the following two sets of key terms: (preschool) AND (“psychostimulants” or “methylphenidate” or “amphetamine”). The keywords and limitations used for different databases are provided in Supplementary Table S1. All articles with titles meeting the inclusion criteria were retrieved and reviewed in full text. All original studies investigating the effects of psychostimulants were eligible for review. Additional eligible studies were identified by examining the reference lists of the primary articles and relevant reviews.

### Inclusion and exclusion criteria

We aimed at determining the therapeutic efficacy of psychostimulants against the symptoms of attention and hyperactivity/impulsivity as well as global symptoms in preschool children with attentional problems. Eligible studies were as follows: (1) RCTs in human, (2) those recruiting preschool children (defined as those ≤6 years of age) who suffered from any attentional or developmental problems, and (3) clinical trials that compared the therapeutic effectiveness against the symptoms of ADHD including inattention, hyperactivity/impulsive and global symptoms between the psychostimulant and control groups. Studies that enrolled children aged equal to or older than 7 years of age, those that were not RCTs, and those that were not in clinical trials were excluded.

### Data extraction and quality assessment

Three investigators (YC Cheng, YS Cheng, and CK Sun) independently extracted relevant information from included studies and evaluated the methodological quality of eligible trials using the Cochrane Collaboration risk of bias tools. Data acquired from the studies included the last name of the first author, year of publication, total number of study participants, type of study, sample size of participant in intervention and control groups, gender, intelligence quotient (IQ), and age as well as psychostimulant dosage and duration. The Cochrane Collaboration’s tool was used to evaluate seven domains of risk: selection bias (sequence generation and concealment), performance bias (blinding of participants and assessors), detection bias (blinding of outcome assessment), attrition bias (incomplete outcome data), selective outcome reporting, and other bias. The risk of bias of each item was rated as low, high, or unclear (if there was insufficient information). Disagreements among the three investigators were resolved through discussion. The overall qualities of evidence for different outcomes were rated according to the Grading of Recommendations Assessment, Development, and Evaluations (GRADE) scoring system [22].

### Efficacy outcomes

The primary outcomes were changes in symptoms of ADHD (i.e., inattention, hyperactivity/ impulsivity, and global symptoms) evaluated by using any clinically validated rating scale from parents’ and teacher’s observation or post-intervention improvements in neuropsychological performance, which were measured by standardized assessment tools such as continuous performance test (CPT). Secondary outcomes were risks of occurrence of adverse events in the psychostimulant group compared with those in the placebo group.

For studies in which relevant data were missing, their authors were contacted by email in an attempt to obtain the necessary information. All potentially relevant manuscripts were independently reviewed by two investigators (YC Cheng and YS Cheng). Areas of disagreement or uncertainty were adjudicated by a third investigator (CK Sun).

### Statistical analysis

To calculate the overall effect size, we calculated the standardized mean differences (SMDs) using the formula for Hedges’ *g* with 95% confidence intervals for continuous outcomes. For dichotomous outcomes, we calculated the odds ratio (OR). Hedges’ *g* is related to Cohen’s *d* and can be interpreted using the same conventions: small (0.2), medium (0.5), and large (0.8) [23]. SMD was calculated to compare the difference in changes in scores after treatment between the psychostimulant and placebo groups. A positive effect size indicated a superior effect of the intervention group in comparison with that in the control group. The standard deviation (SD) of the changes in scores from baseline was calculated using the formula: SD = square root [(SD pretreatment) ^2^ + (SD post-treatment) ^2^ − (2R × SD pretreatment × SD post-treatment)], assuming a correlation coefficient (*R*) = 0.5, when the studies did not report them. When only the standard error of the mean (SEM) was reported, SD was calculated by multiplying the SEM by the square root of the sample size. For studies using median and range, mean and SD were estimated using the formula according to the Cochrane guidelines [24, 25]. In studies that used multiple doses of psychostimulants, we combined the means and SDs for the different dosage groups to give single values for the intervention group. For adverse effects presented as binary outcomes, the effect size was calculated using the OR.

The degree of heterogeneity or inconsistency across the included trials was assessed with *I*^2^ test [26]. To offer more generalizable results, a random effects model was used on the assumption of a variation in true effect size. Publication bias was examined using a funnel plot and Egger’s regression test was used if there were more than 10 datasets [27]. Leave-one-study-out sensitivity analysis was performed by the sequential exclusion of one trial at a time to examine the robustness of the pooled effects. To explore the potential effect of trial-level modifiers, we considered several covariates in meta-regression approaches including mean age, mean daily dosage of methylphenidate, female proportion, treatment duration, and IQ. All meta-analytic computations were performed with the R software (R x64 version 4.1.2, The Cochrane Collaboration, Oxford, UK).

## Results

### Baseline characteristics of included studies

**Figure 1.**
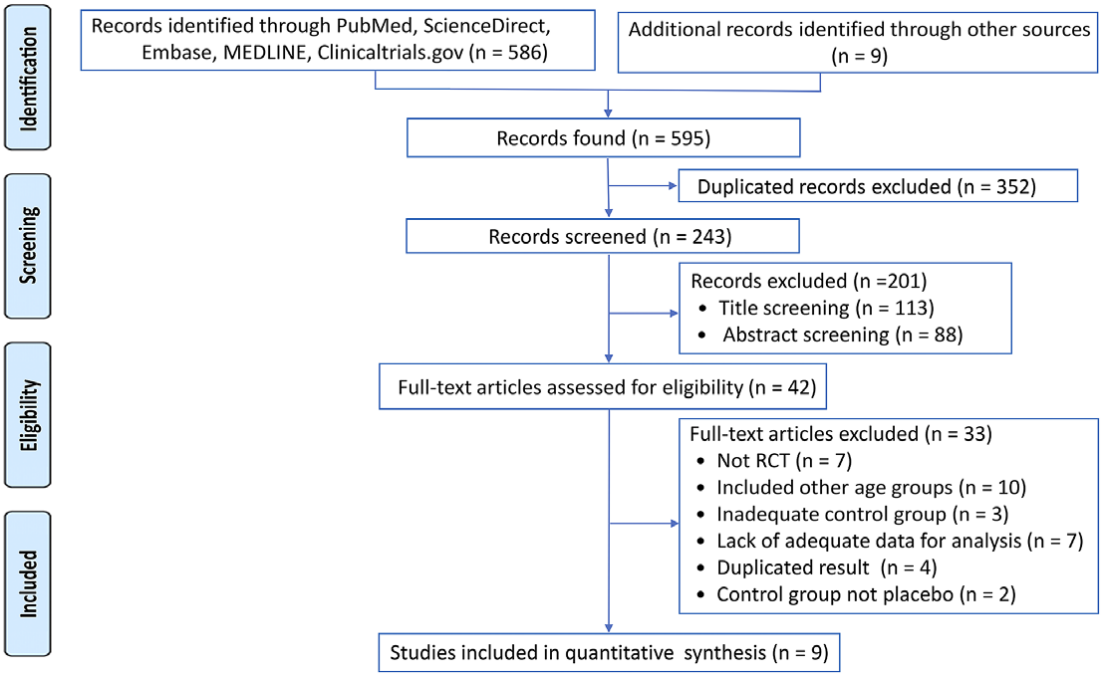
PRISMA diagram of identifying eligible studies.

Figure 1 is a flowchart summarizing the review process in accordance with the PRISMA statement [28]. Of the 595 original studies screened, nine met the inclusion criteria for qualitative synthesis [29–37]. The reasons for study exclusions are provided in Supplementary Table S2. A summary of the characteristics of the nine included studies for qualitative synthesis and their results are presented in Table 1. The population size of the included studies ranged from 28 to 330 with a total of 544 being preschool children (mean age = 4.86 years, female proportion = 11.98%, median treatment duration = 4.33 weeks). There was a wide variation in dosing strategies across the included studies, including a fixed dose based on body weight, a fixed daily dose, or the best dose approach, instead of reporting a mean dose or dose range. Details on the dosage used in the included studies are summarized in Table 1. The duration of intervention across the included trials was between one and 6 weeks. The results of quality assessment of our included trials assessed by the authors based on the seven domains of risk described in the Cochrane Collaboration tool are presented in Supplementary Figure S1, S2. Overall, most studies had a fair quality in double blinding but did not report clear procedures about their randomization process. Many studies are given a high risk of bias in other biases because they used placebo lead-in [32], safety lead-in [30, 34], or excluded participants who failed to respond or tolerate psychostimulants [31, 32].

**Table 1.** Summary of characteristics of included studies in the current meta-analysis.

References	Diagnosis (criteria)	Exclusion criteria	Design	Comparison	Dosage(mean)	*N*	Duration (weeks)	Outcome	IQ	Subtype	Age(years)	Female(%)	Country
[31]	1. ADHD(DSM-5) 2. IQ equivalent of >80	A lack of response to a trial of adequate dose and duration of MPH and intolerance to previous MPH treatment; were receiving psychotropic medication	RCT	MPH-MLR	Best dose (27.5 mg/d)	40	2	Parent: ADHD-RS-IV total score	N/A	Combined 81.1% Inattention 7.6% H/I 11.3%	4.9 (4–5.7)	23.4	U.S.
				Placebo	None	50							
[32]	1. AD, Asperger disorder, or PDD-NOS (DSM-IV-TR) or DD 2. symptoms of hyperactivity and impulsivity in multiple settings	Prior failed treatment with MPH; concurrent medications having central nervous system effects; history of tics; major medical condition that could be affected negatively by MPH; and diagnosis of bipolar disorder, psychosis, significant suicidality, or other psychiatric disorders requiring treatment with additional medication.	RCT/ Cross-over	MPH IR	Best dose (14.46 mg/d)	14	4	Parent: CPRS-r DSM-IV-ADHD Subscale and Nisonger Child Behavior Rating Form-Parent-Hyperactive Subscale	74.9	N/A	4.8 (3–5.9)	7.1	U.S.
				Placebo	None	14							
[34]	1. ADHD (DSM-IV) 2. IQ equivalent of >70	Adjustment disorder, pervasive developmental disorders, psychosis, significant suicidality, or other psychiatric disorder in addition to ADHD that required treatment with additional medication; current stimulant or cocaine abuse in a relative living in the home; a confounding medical condition; inability of the parent to understand or follow study instructions, or history of bipolar disorder in both biological parents.	RCT	MPH IR	Best dose	61	4	Parent: SWAN total, inattention, hyperactivity/impulsivity Teacher: SWAN total, inattention, hyperactivity/impulsivity	97.41	Combined 74.6% H/I 25.4%	4.42 (3–5.5)	25.4	U.S.
				Placebo	None	53							
[30]	1. ADHD (DSM-IV) 2. IQ equivalent of >70	Adjustment disorder, pervasive developmental disorders, psychosis, significant suicidality, or other psychiatric disorder in addition to ADHD that required treatment with additional medication; current stimulant or cocaine abuse in a relative living in the home; a confounding medical condition; inability of the parent to understand or follow study instructions, or history of bipolar disorder in both biological parents.	RCT/ Cross-over	MPH IR	1.5 mg tid	165	1	Parent: CLAM/SKAMP composite, SKAMP Attention and SKAMP Deportment Teacher: CLAM/SKAMP composite, SKAMP Attention and SKAMP Deportment	97.93	Combined 75% H/I 25%	4.74 (3–5.5)	26	U.S.
				MPH IR	2.5 mg tid								
				MPH IR	5 mg tid								
				MPH IR	7.5 mg tid								
				Placebo	None	165							
[35]	ADHD (DSM-llI-R or DSM-IV)	N/A	RCT/ Cross-over	MPH IR	0.3 mg/kg bid	36	6	Teacher: point system - Percentage following activity rules	102	N/A	6.13 (5–6)	11	U.S.
				MPH IR	0.6 mg/kg bid	36							
				Placebo	None	36							
[29]	ADHD (DSM-IV)	N/A	RCT/ Cross-over	MPH IR or MAS	Best dose	28	3 or 4	Parent: ASQ Teacher: ASQ	N/A	N/A	5.25 (3–5)	15	U.S.
				Placebo	None	28							
[36]	ADHD (DSM-llI-R)	Autism/pervasive development disorder	RCT/ Cross-over	MPH IR	0.3 mg/kg bid	11	3	Teacher: CTRS inattention and hyperactivity	60	N/A	4.91 (4–5.11)	18.1	U.S.
				MPH IR	0.6 mg/kg bid	11							
				Placebo	None	11							
[33]	1. ADHD (DSM-llI-R) 2. >80 Peabody Picture Vocabulary Test	Sensory or physical handicaps. Developmental disorders (e.g., autism), neurological disease, or obvious CNS dysfunction as assessed by a pediatrician.	RCT/ Cross-over	MPH IR	0.3 mg/kg bid	31	1	Parent: CPRS Learning, conduct and hyperactivity index Cognitive tasks: Gordon Delay Efficiency ratio and Gordon Vigilance Commission errors	N/A	N/A	4.89 (4–5.83)	16.1	Canada
				MPH IR	0.5 mg/kg bid	31							
				Placebo	None	31							
[37]	1. Minimal brain dysfunction 2. Not retarded	Gross sensory pathology, seizure disorder, and family psychopathology	RCT	MPH IR	Best dose	29	6	CPT: omission and commission	N/A	N/A	4.81 (<6)	25.4	U.S.
				Placebo	None	26							

Abbreviations: AD, autistic disorder; ADHD, attention-deficit/hyperactivity disorder; ADHD-RS-IV, attention-deficit/hyperactivity disorder rating scale-IV; ASQ, abbreviated symptoms questionnaire; bid, twice a day; CLAM, Conners, Loney, and Milich rating scales; CNS, central nervous system; CPRS, conners parent rating scale; CPRS-r DSM-IV-ADHD, conners parent rating scales–revised DSM-IV-ADHD; CPT, continuous performance test; CTRS, conners teacher rating scale; d, day; DD, developmental delay; DSM-5, the diagnostic and statistical manual of mental disorders, fifth edition; DSM-llI-R, diagnostic and statistical manual of mental disorders, third edition, revised; DSM-IV, diagnostic and statistical manual of mental disorders, fourth edition; DSM-IV-TR, diagnostic and statistical manual of mental disorders, fourth edition, text revision; IQ, intelligence quotient; H/I, hyperactivity/impulsivity; MAS, mixed amphetamine salts; MPH, methylphenidate; MPH IR, methylphenidate immediate release; MPH-MLR, extended-release methylphenidate; N, number, N/A, not available; RCT, randomized controlled trial; PCIT, parent child interaction therapy; PDD-NOS, pervasive developmental disorder not otherwise specified; SKAMP, Swanson, Kotkin, Atkins, M-Flynn, and Pelham rating scale; SWAN, strengths and weaknesses of attention-deficit/hyperactivity disorder symptoms and normal behavior scale; tid, three times a day.

### Pooled effects of psychostimulant treatment on global ADHD symptoms

**Figure 2.**
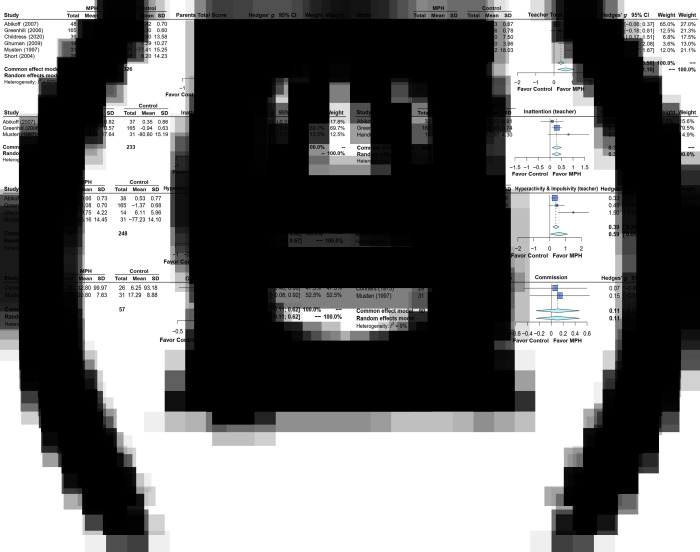
Forest plots of effect sizes for comparing the difference in the improvement of ADHD symptoms between psychostimulant and placebo groups: (A) global symptoms from parents’ observation, (B) global symptoms from teachers’ observation; (C) symptoms of inattention from parents’ observation, (D) symptoms of inattention from teachers’ observation; (E) symptoms of hyperactivity/impulsivity from parents’ observation, (F) symptoms of hyperactivity/impulsivity from teachers’ observation; (G) omission, and (H) commission. *A positive effect size indicated a superior effect of the intervention group in comparison with that in the control group. CI confidence interval; MPH methylphenidate; SD standard deviation.

The results of meta-analysis of six trials involving are summarized in Table 2. Our findings showed better treatment efficacy of psychostimulants than placebo for global symptoms of ADHD from parent’s observation in preschool children (Hedges’ *g* = 0.6152, 95% CI [0.3228; 0.9075], *p* < 0.0001, *I^2^* = 57.1%) (Figure 2A). With respect to improvements in global symptoms of ADHD from teacher’s observation, our results also demonstrated significantly better treatment efficacy of psychostimulants than placebo (Hedges’ *g* = 0.6563, 95% CI [0.2110; 1.1016], *p* = 0.0039, *I^2^* = 78.2%) (Figure 2B). Visual inspection of the funnel plot revealed some degree of asymmetry (Supplementary Figure S3a,b).

**Table 2.** Effect sizes for comparing the difference in the improvement of ADHD symptoms between psychostimulants and placebo groups.

Domains	Rating scale or tests	Number of studies	Patients/controls	Effect sizes (95%CI)	Effect size (*p* value)	Heterogeneity *I*^2^ (%)
Total score (parents)	1. ADHD-RS-IV total score; 2. CPRS-r DSM-IV-ADHD Subscale and Nisonger Child Behavior Rating Form-Parent-Hyperactive Subscale; 3. SWAN total; 4. CLAM/SKAMP composite; 5. ASQ; 6. CPRS Learning, conduct and hyperactivity index	6	325/326	0.6152 (0.3228; 0.9075)	<0.0001***	57.1%
Total score (teachers)	1. SWAN total; 2. CLAM/SKAMP composite; 3. Point system - Percentage following activity rules; 4. ASQ; 5. CTRS inattention and hyperactivity	5	263/263	0.6563 (0.2110; 1.1016)	0.0039***	78.2%
Inattention (parents)	1. SWAN inattention; 2. SKAMP Attention; 3. CPRS Learning	3	244/233	0.3268 (0.1455; 0.5080)	0.0004***	27.8%
Inattention (teachers)	1. SWAN inattention; 2.SKAMP Attention; 3. CTRS inattention	3	208/208	0.2950 (0.1015; 0.4886)	0.0028***	0.0%
Hyperactivity/impulsivity (parents)	1. SWAN hyperactivity/impulsivity; 2. SKAMP Deportment; 3. Nisonger Child Behavior Rating Form-Parent-Hyperactive Subscale; 4. CPRS hyperactivity index	4	257/248	0.4510 (0.2293; 0.6727)	<0.0001***	27.5%
Hyperactivity & impulsivity(teacher)	1. SWAN hyperactivity/impulsivity; 2. SKAMP Deportment; 3. CPRS hyperactivity index	3	208/209	0.5933 (0.0539; 1.1327)	0.0311***	63.4%
Omission	1. CPT; 2. Gordon Delay Efficiency ratio	2	60/57	0.2518 (−0.1131; 0.6168)	0.1762	0.0%
Commission	1. CPT; 2. Gordon Vigilance Commission errors	2	60/57	0.1097 (−0.2533; 0.4727)	0.5536	0.0%

Abbreviations: ADHD-RS-IV, attention-deficit/hyperactivity disorder rating scale-IV; CLAM, Conners, Loney, and Milich rating scales; CPRS-r DSM-IV-ADHD, conners parent rating scales–revised DSM-IV-ADHD; CPT, continuous performance test; CTRS, conners teacher rating scale; SWAN, strengths and weaknesses of attention-deficit/hyperactivity disorder symptoms and normal behavior scale; SKAMP, Swanson, Kotkin, Atkins, M-Flynn, and Pelham rating scale; ASQ, abbreviated symptoms questionnaire; CPRS, conners parent rating scale.

p<0.05 statistical singificance ***

### Efficacy of psychostimulants against inattention

Analysis of inattentive symptoms showed a significantly better treatment effect of psychostimulants compared with that of placebo from both parents’ (*n* = 3, Hedges’ *g* = 0.3268, 95% CI [0.1455; 0.5080], *p* = 0.0004, I^2^ = 27.8%) (Figure 2C) and teacher’s observations (*n* = 5, Hedges’ *g* = 0.2950, 95% CI [0.1015; 0.4886], *p* = 0.0028, I^2^ = 0.0%) (Figure 2D). Visual inspection of funnel plot revealed symmetry (Supplementary Figure S3c,d).

### Efficacy of psychostimulants against hyperactivity and impulsivity

Analysis of four studies focusing on the effect of psychostimulants against the symptoms of hyperactivity/impulsivity revealed a significantly better therapeutic effect of psychostimulants than that of placebo from parents’ observation (Hedges’ *g* = 0.4510, 95% CI [0.2293; 0.6727], *p* < 0.0001, *I^2^* = 27.5%) (Figure 2E). In addition, the results from three studies investigating the effect of psychostimulants on the symptoms of hyperactivity and impulsivity demonstrated a significant beneficial effect from teacher’s observation (Hedges’ *g* = 0.5933, 95% CI [0.0539; 1.1327], *p* = 0.0311; I^2^ = 63.4%) (Figure 2F). Visual inspection of the funnel plot revealed symmetry in results from parents’ observation but some degree of asymmetry in results from teachers’ observation (Supplementary Figure S3e,f).

### Efficacy of psychostimulants against omission and commission

Only two out of the nine included studies investigated the effect of psychostimulants on attentional performance including omission and commission by using standardized computerized tests [33, 37]. While one study used a CPT [37], the other adopted the Gordon Diagnostic System (GDS) [33]. Our results showed that the therapeutic effect of psychostimulants was not significantly better than that of placebo on omission (Hedges’ *g* = 0.2518, 95% CI [−0.1131; 0.6168], *p* = 0.1762, I^2^ = 0.0%) (Figure 2G) and commission (Hedges’ *g* = 0.1097, 95% CI [−0.2533; 0.4727], *p* = 0.5536, I^2^ = 0.0%) (Figure 2H).Visual inspection of the funnel plot revealed symmetry (Supplementary Figure S3g,h).

### Secondary analysis of risk of side effects associated with psychostimulant treatment

A comparison of the occurrence of adverse events between the psychostimulant and placebo groups demonstrated that only the risk of decreased appetite was significantly higher in the psychostimulant group than that in the placebo group (*n* = 6, OR = 2.3899 [1.0807; 5.2852], *p* = 0.0314). Although the risks of irritability, sleep disturbance, prone to cry, and anxiety were slightly higher in preschool children receiving psychostimulant treatment than those treated with placebos (OR = 1.06, 1.312, 1.359, and 1.318, respectively), none of these elevated risks reached statistical significance (Supplementary Table S3a). Analysis of the three studies with available information about blood pressure and heart rate showed no significant difference in these parameters between the psychostimulant and placebo groups (Supplementary Table S3b). The common side effects described in the included studies are summarized in Supplementary Table S4.

### Robustness of evidence and quality of evidence

An examination of the robustness of the outcomes from the current meta-analyses with a leave-one-out sensitivity analysis revealed inconsistencies in results regarding the symptoms of inattention from parents’ observation, the symptoms of inattention from teachers’ observation, and the symptoms of hyperactivity/Impulsivity from teachers’ observation, suggesting a tentative nature of these findings (Supplementary Table S5). GRADE assessment revealed that the qualities of evidence regarding the study outcomes ranged from low to very low, mainly due to problems of inconsistency and imprecision of study results (Supplementary Table S6).

### Factors associated with improvements in global symptoms

Our meta-regression analysis demonstrated a positive association between an older age and a better improvement in the global symptoms of ADHD from parents’ observation (Table 3a). Similarly, improvements in the global symptoms of ADHD were found to be positively associated with an older age and a longer duration of treatment but negatively related to female proportion from a teacher’s perspective (Table 3b). Therefore, our results suggested a better efficacy of psychostimulants in older individuals or in those treated for a longer duration. On the other hand, the efficacy of psychostimulants was poorer in studies that included more females, implying that the observed efficacy of psychostimulants may be less obvious in female participants.

**Table 3.** Regression coefficients of correlations between continuous variables and improvement in (a) parents total score and (b) teacher total score, in included studies using mixed-effects model.

(a) Parents total scores				
		Meta-regression		
Covariate	No. of studies	ß (95% CI)	*p* value	Proportion of variance explained (%)
Dose	4	−0.0034 (−0.0289; 0.0222)	0.7957	0.00
Age	5	1.2716 (0.4456; 2.0977)	0.0026	100.00
Female proportion	5	−0.0331 (−0.0870; 0.0218)	0.2378	31.04
Duration	5	0.0105 (−0.3310; 0.3523)	0.9518	0.00

(b) Teacher total scores				
		Meta-regression		
Covariate	No. of studies	ß (95% CI)	*p* value	Proportion of variance explained (%)
Age	4	0.5648 (0.0012; 1.1284)	0.0495*	66.48
Female proportion	5	−0.0682 (−0.1005; −0.0360)	<0.0001*	100.00
IQ	4	−0.0130 (−0.0520; 0.0259)	0.5119	0.00
Duration	5	0.1821 (0.0321; 0.3321)	0.0174*	76.12

* ß, standardized beta coefficient for meta-regression.

## Discussion

Although the therapeutic effectiveness of psychostimulants against symptoms of ADHD has been well-documented in children/adolescents as well as the adult population in previous systematic reviews [8, 38, 39], none of them focused particularly on preschool children. To our best knowledge, the present meta-analysis, which included nine RCTs and 544 participants, was the first to demonstrate the effectiveness of psychostimulants against the symptoms of ADHD in preschool children. We further found that not only were the observed treatment efficacies of psychostimulants similar between parents and teachers but psychostimulants also seemed to be more effective for symptoms of hyperactivity/impulsivity than inattention in this population. Moreover, our meta-regression analysis showed that a longer treatment duration, older age, and male gender were associated with better treatment efficacy.

With respect to our primary outcomes, our findings in support of the therapeutic efficacy of psychostimulants for both core symptoms of ADHD (i.e., inattention and hyperactivity/impulsivity) as well as the global symptoms of ADHD indicated that the use of psychostimulants may improve not only the core symptoms of ADHD but also the general behaviors of ADHD children. Nevertheless, sensitivity analysis revealed a loss of significant therapeutic effectiveness against the symptoms of inattention from parents’ observation, the symptoms of inattention from teachers’ observation, and the symptoms of hyperactivity/Impulsivity from teachers’ observation after removal of the trial by Greenhill [30], suggesting that more studies are required to support the therapeutic efficacy of psychostimulants for these symptoms. Compared with the results of a recent network meta-analysis focusing on other age groups [8], the effect size of treatment efficacy of psychostimulants for global symptoms of ADHD in preschool children was slightly smaller than that in school-aged children (ES = 0.66 versus 0.78, respectively), but similar to that in adult patients (ES = 0.66 versus 0.49 to 0.79 for global symptoms, respectively). However, in contrast to this study that had a relatively small sample size (*n* = 544), the effect sizes of the previous network meta-analysis were derived from large populations of adults (*n* = 10,296) and children/adolescents (*n* = 14,342). Moreover, the qualities of current evidence supporting the use of psychostimulants in this population ranged from low to very low, mainly due to inconsistency and imprecision of study outcomes, as well as limited sample sizes. Therefore, although our results support the use of psychostimulants against the symptoms of ADHD in preschool children, further studies are still needed to provide more tangible evidence.

Despite our finding of moderate treatment efficacy regarding the use of psychostimulants for treating the symptoms of hyperactivity/impulsivity, the effect size was small for the symptom of inattention. Because the symptoms of hyperactivity/impulsivity among preschool children are school teachers’ major concerns that can be readily identified [13], under-reporting of such symptoms from teachers is unlikely. Consistently, our results showed a slightly larger effect size of treatment efficacy for hyperactivity/impulsivity from a teacher’s perspective than that from parents’ observation (ES = 0.59 versus 0.3, respectively). In contrast, inattention was more difficult to identify in preschool children [13]; therefore, it is important to be aware that symptom improvements based on raters’ observations may not reliably reflect the changes in underlying neuropsychological functioning. Of the nine included RCTs, only two provided information about the objective attentional performance of the recruited preschool children independent of observers’ judgment. Interestingly, the effect size for commission, which may be more related to impulsivity, was smaller than the effect size for omission, which may be more representative of attentional functioning. Nevertheless, none of them achieved statistical significance probably due to their small sample sizes. Therefore, although psychostimulants seemed more effective for hyperactivity/impulsivity in this study, more studies especially those including neuropsychological testing that involves a more objective assessment of different aspects of attentional functions [40], are required to shed light on the mechanisms underlying the observed improvement in behavioral problems.

Our meta-regression further showed that age and gender differences were both associated with the treatment efficacy of psychostimulants against the global symptoms of ADHD. Taking into account that the higher prevalence of hyperactivity and behavioral disturbances among male ADHD children than among their female counterparts in classes [41], behavioral improvements in hyperactivity/impulsivity may be more easily observed in male preschool children. Besides, the efficacy of psychostimulants may be more easily observed in older age groups because inattention is likely to be ignored in preschool children until they enter a more structured learning environment as they get older [13]. Finally, our meta-regression finding of an association between a longer duration of treatment and better therapeutic effects of psychostimulants may suggest a benefit of a prolonged treatment for those who exhibited an unsatisfactory response to initial therapy.

Safety issues and potential adverse effects were the most important concerns regarding the use of psychostimulants in preschool children [30]. Limited information available in our included trials precluded the conduction of an analysis of the overall dropout rates or dropouts due to adverse events. Nevertheless, our secondary analysis identified the risk of decreased appetite as the only side effect that was significantly related to psychostimulant use, while the risks of other side effects including irritability, sleep disturbance, proneness to cry, and anxiety were only slightly and non-significantly higher in the psychostimulant group than in the placebo group (OR all <1.5). In addition, despite the reported hemodynamic impacts of blood pressure and heart rate elevations associated with psychostimulant use [42], our results did not demonstrate significant differences in these parameters between the psychostimulant treatment and placebo groups. Overall, psychostimulants seemed well-tolerated in general despite the significant correlation with the risk of decreased appetite in this age group. However, given the possible risks of side effects such as a decrease in appetite and potential influence on heart rate and blood pressure [42], and low quality of current evidence ranging from low to very low in GRADE assessment, judicious prescription of psychostimulants for preschool children is recommended.

There are several limitations in the present meta-analysis. First, the inclusion of only nine RCTs with 544 participants may preclude an accurate assessment of studies outcomes. In particular, given the limited number of included trials, publication bias was assessed only by visual inspection of the funnel plot rather than analyzed by Egger’s test. In addition, imprecisions and inconsistencies of study results together with the small sample sizes may contribute to the low to very low qualities of evidence derived from the current meta-analysis. Moreover, our sensitivity analysis further revealed a significant impact of one study [30] on the overall results about the symptoms of inattention from both teachers’ and parents’ observations, as well as hyperactivity/impulsivity from a teacher’s perspective. Therefore, more large-scale RCTs are required for verification of our study outcomes. Second, most of the included studies did not provide clear detail about their randomization processes, and authors in six out of the nine studies received some financial support from pharmaceutical companies [29–32, 34, 35]. Moreover, most studies adopted a cross-over design without washout periods between placebo and psychostimulant interventions [29, 30, 32, 33, 35, 36]. Nevertheless, most psychostimulants had a short half-life [43] that may not bias our findings. Third, some studies used placebo lead-in [32] or safety lead-in [30, 34] designs, and two other studies excluded participants who either failed to respond or could not tolerate psychostimulant treatment in the past [31, 32]. Therefore, it is possible that these studies may underestimate the occurrence of side effects and over-estimate the observed clinical effectiveness of psychostimulants. Fourth, the wide variations in doses and prescription strategies of psychostimulants (that is fixed dose, dosage based on body weight or flexible dose) across the included studies precluded a subgroup analysis to clarify a dose–response relationship in efficacy and also side effects of psychostimulants in the study populations. Fifth, heterogeneity arising from the use of different behavioral rating scales and exclusion of autism or intellectual functioning in some of the trials may also bias our results. Sixth, only six out of the 11 included studies provided information about side effect profiles and categorization (e.g., irritability or emotional lability). Moreover, because the approaches to side effect categorization were not unified across those studies, there was a potential risk of under- or over-reporting of certain side effects. Finally, all trials were conducted in North America; therefore, our results may not be ethnically or geographically extrapolated to other countries.

## Conclusion

In summary, our results supported the effectiveness of psychostimulants against the symptoms of ADHD in preschool children, especially for hyperactivity/impulsivity from teachers’ observation. Better therapeutic effects of psychostimulants were observed in those with an older age, male gender, and a longer duration of treatment. Psychostimulants also seemed to have tolerable side effect profiles for most preschool children. However, given the risks of potentially severe adverse effects and limited quality of the current evidence, more research is warranted to support their use in this age group.

## Data Availability

Statement Data that supports the findings of this study are available from the corresponding author upon request.

## Supplementary material

For supplementary material accompanying this paper visit http://doi.org/10.1192/j.eurpsy.2023.11. click here to view supplementary material
