# Supplementary material for: Efficacy and tolerability of psychostimulants for symptoms of attention-deficit hyperactivity disorder in preschool children: A systematic review and meta-analysis
Source: Eur Psychiatry. 2023 Feb 15;66(1):e24. doi: 10.1192/j.eurpsy.2023.11 (PMC10044299; doi:10.1192/j.eurpsy.2023.11)
Supplement: Supplementary file 1 [file S0924933823000111sup001.zip › S0924933823000111sup003.pptx]

## Slide 1
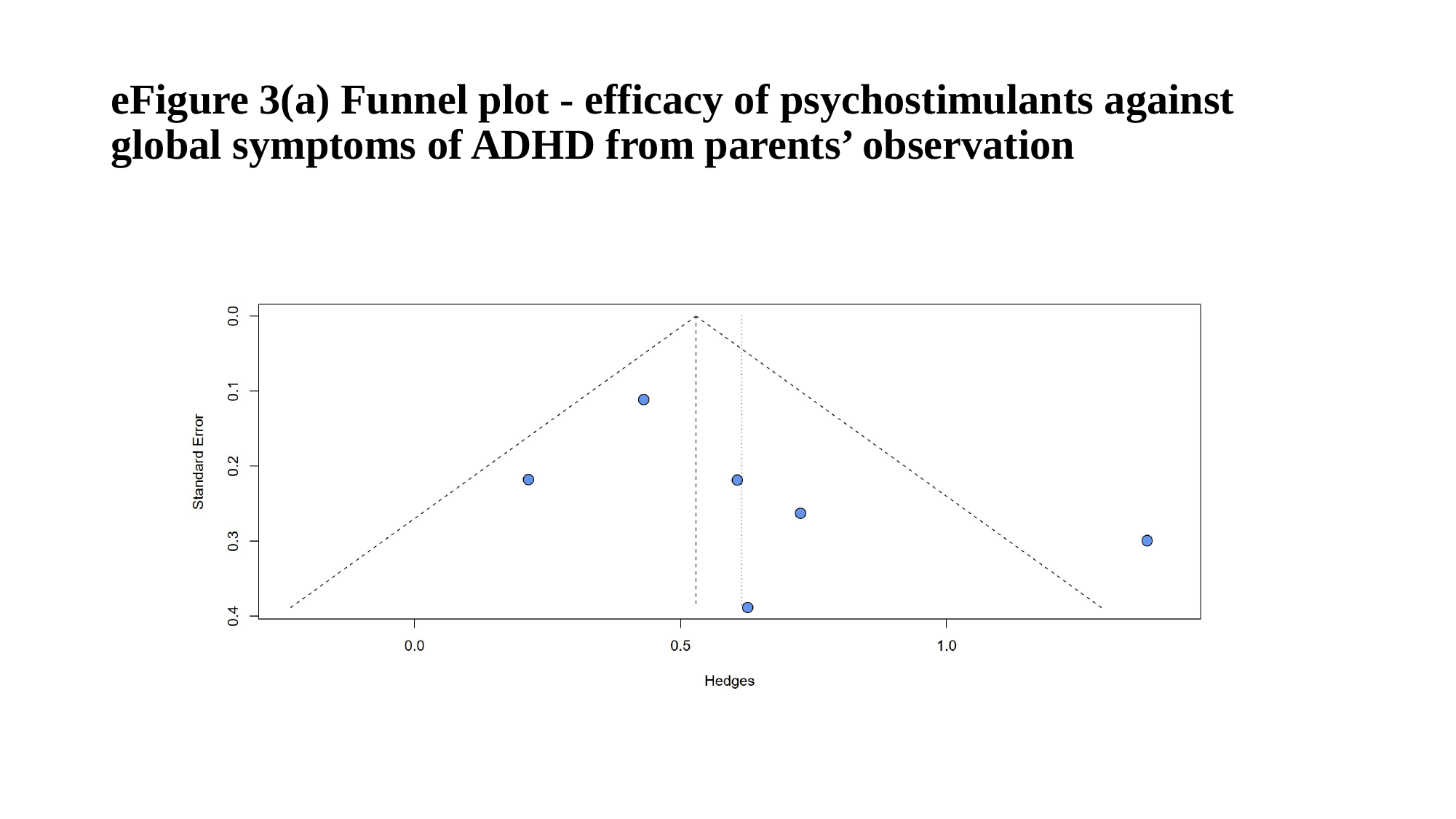

# eFigure 3(a) Funnel plot - efficacy of psychostimulants against global symptoms of ADHD from parents’ observation

## Slide 2
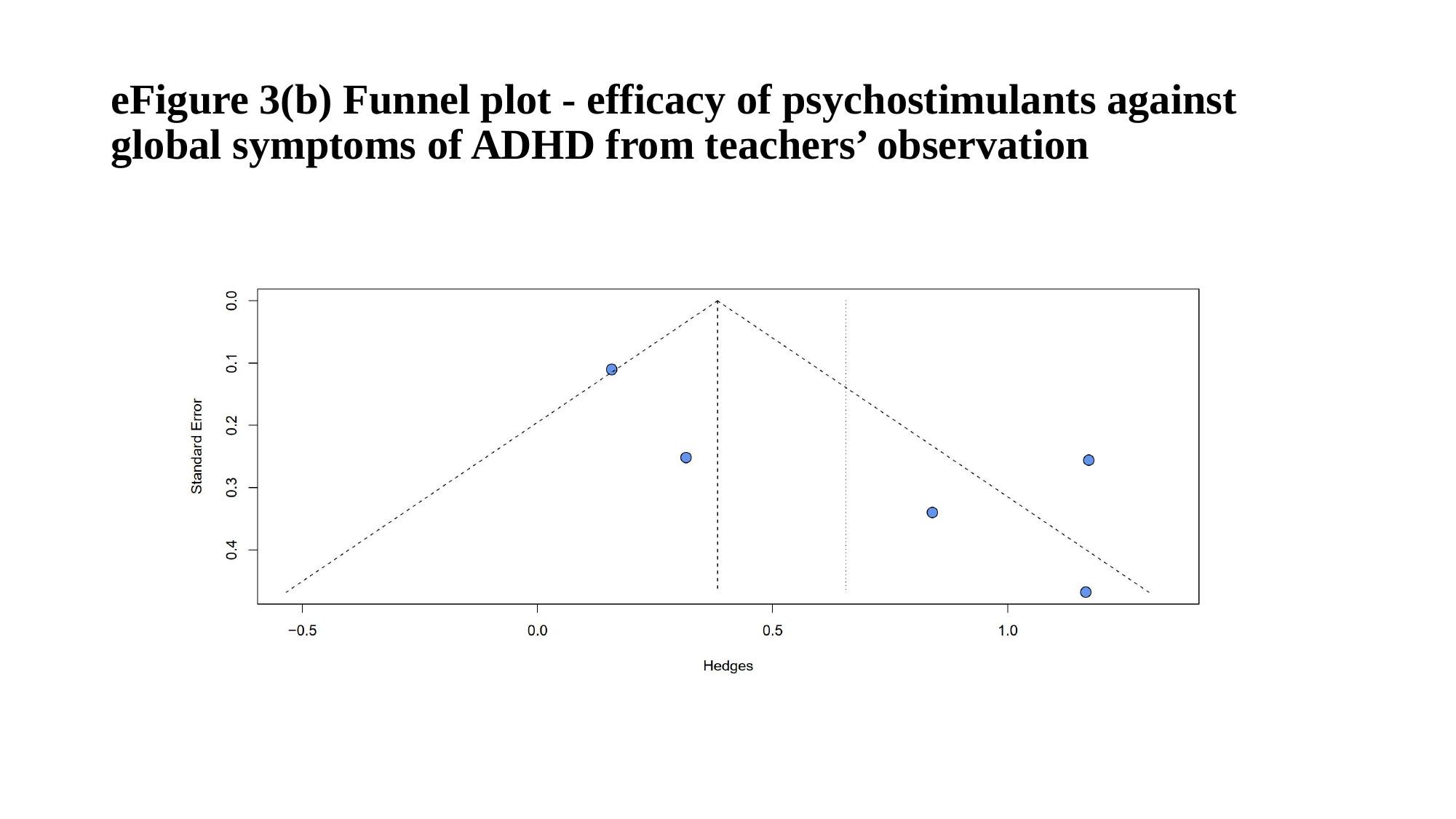

# eFigure 3(b) Funnel plot - efficacy of psychostimulants against global symptoms of ADHD from teachers’ observation

## Slide 3
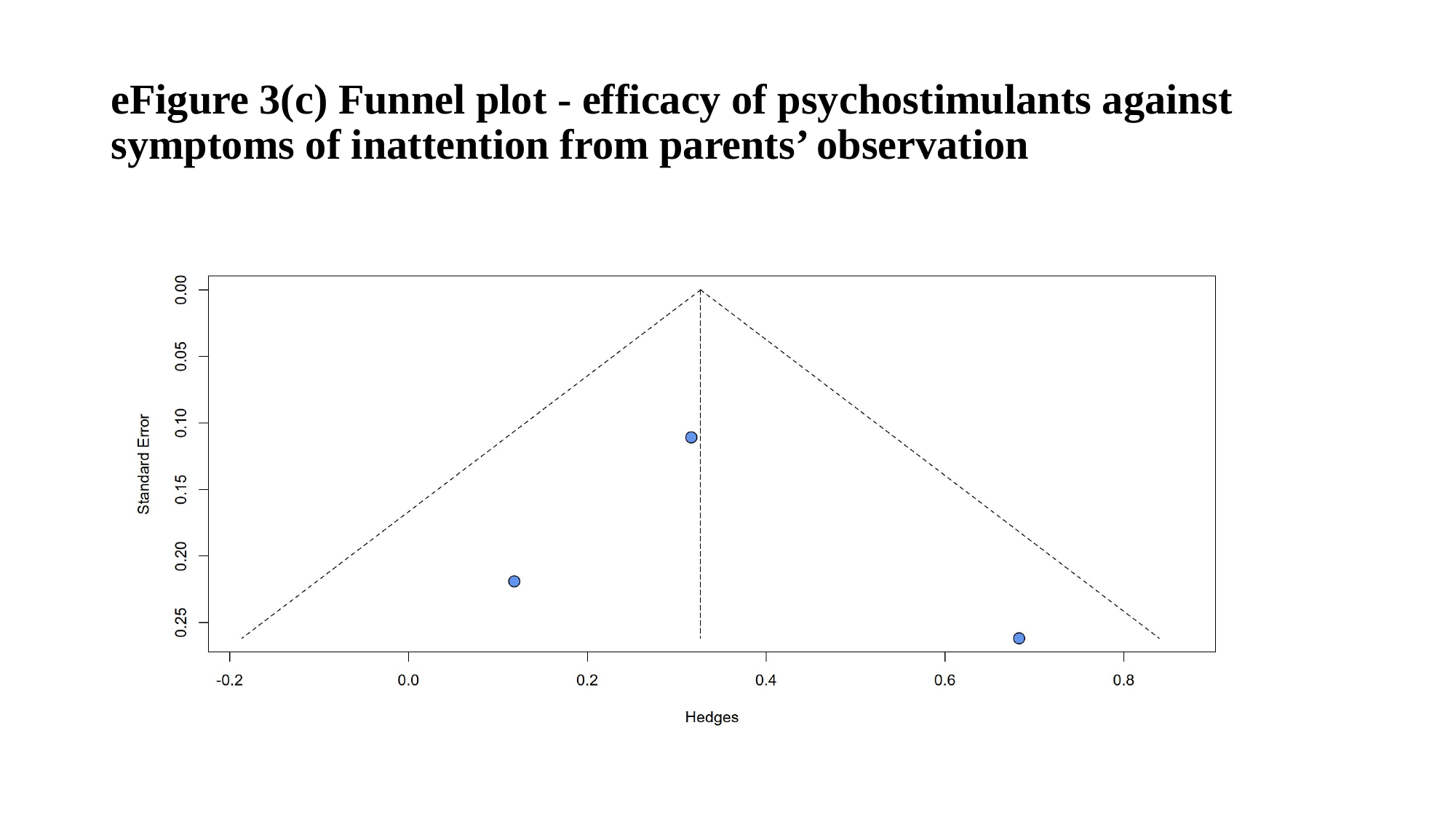

# eFigure 3(c) Funnel plot - efficacy of psychostimulants against symptoms of inattention from parents’ observation

## Slide 4
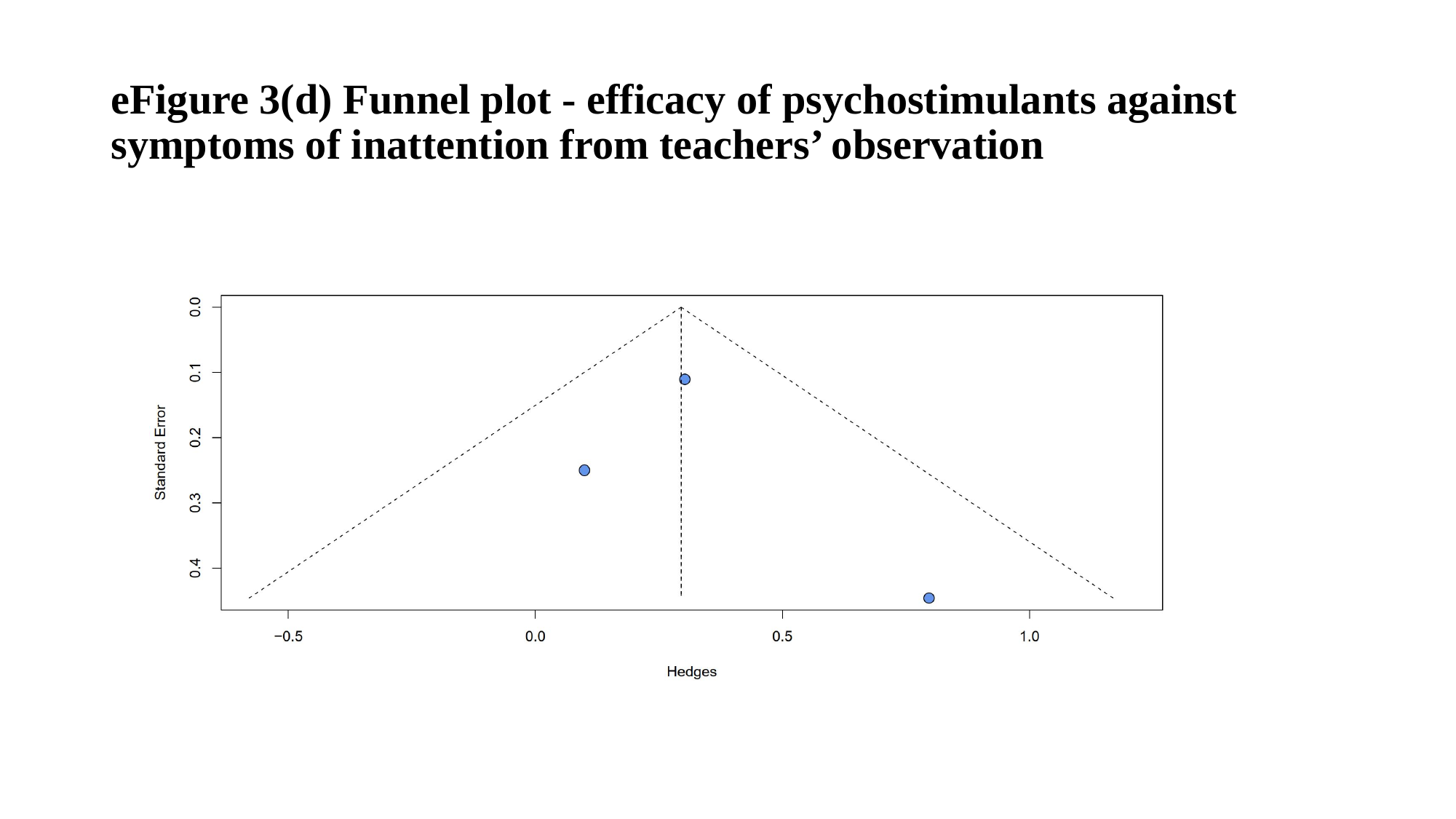

# eFigure 3(d) Funnel plot - efficacy of psychostimulants against symptoms of inattention from teachers’ observation

## Slide 5
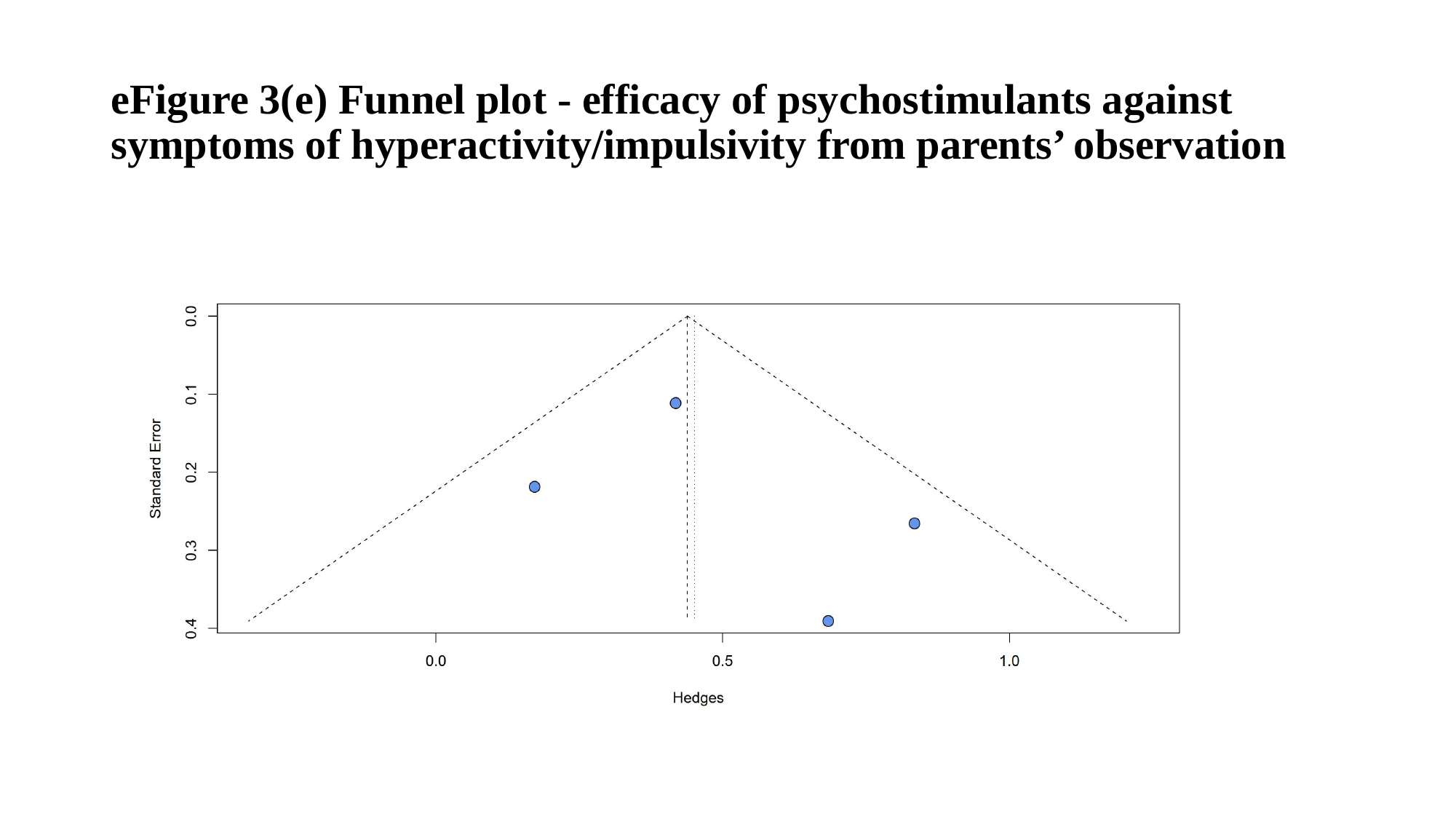

# eFigure 3(e) Funnel plot - efficacy of psychostimulants against symptoms of hyperactivity/impulsivity from parents’ observation

## Slide 6
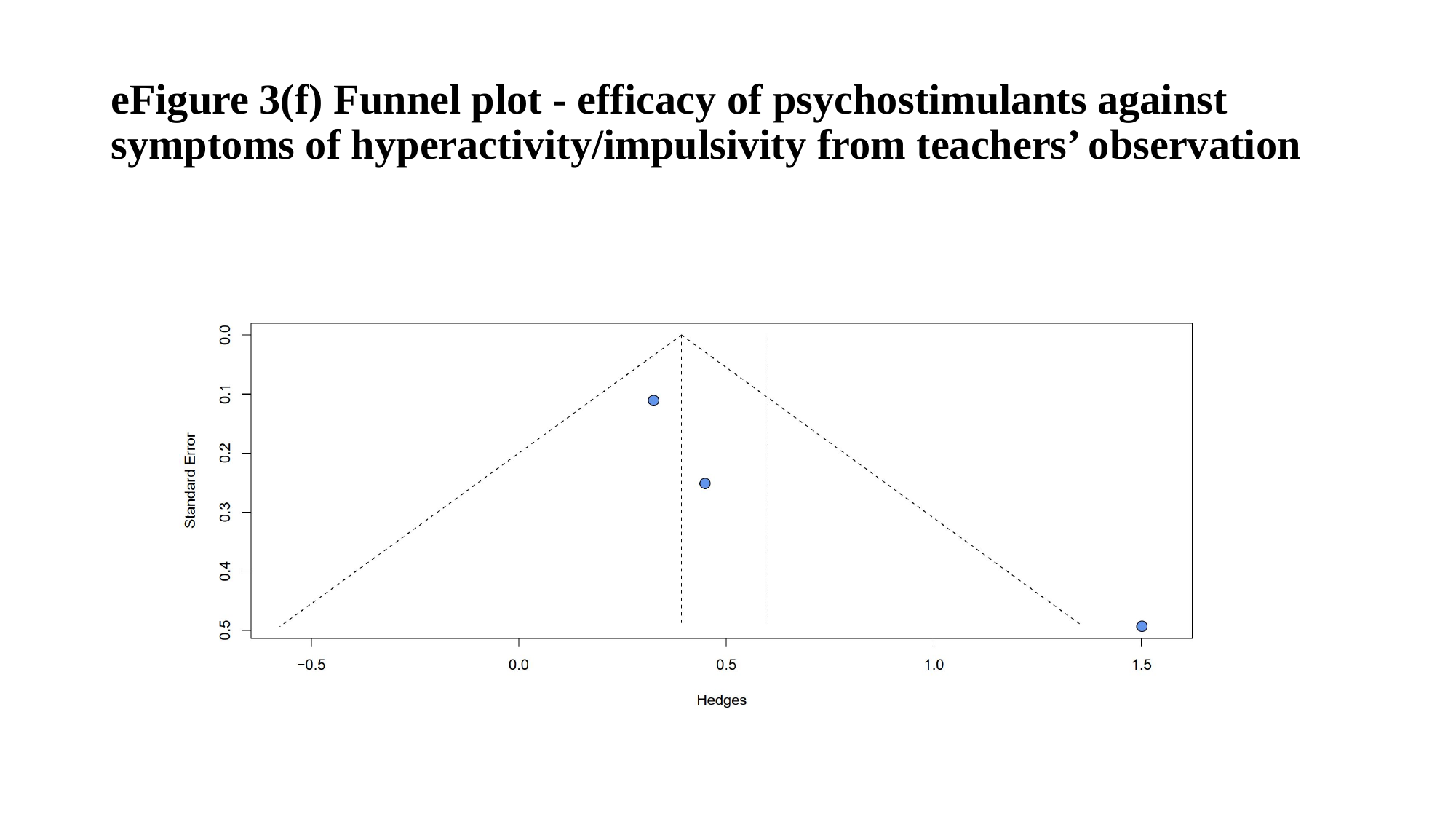

# eFigure 3(f) Funnel plot - efficacy of psychostimulants against symptoms of hyperactivity/impulsivity from teachers’ observation

## Slide 7
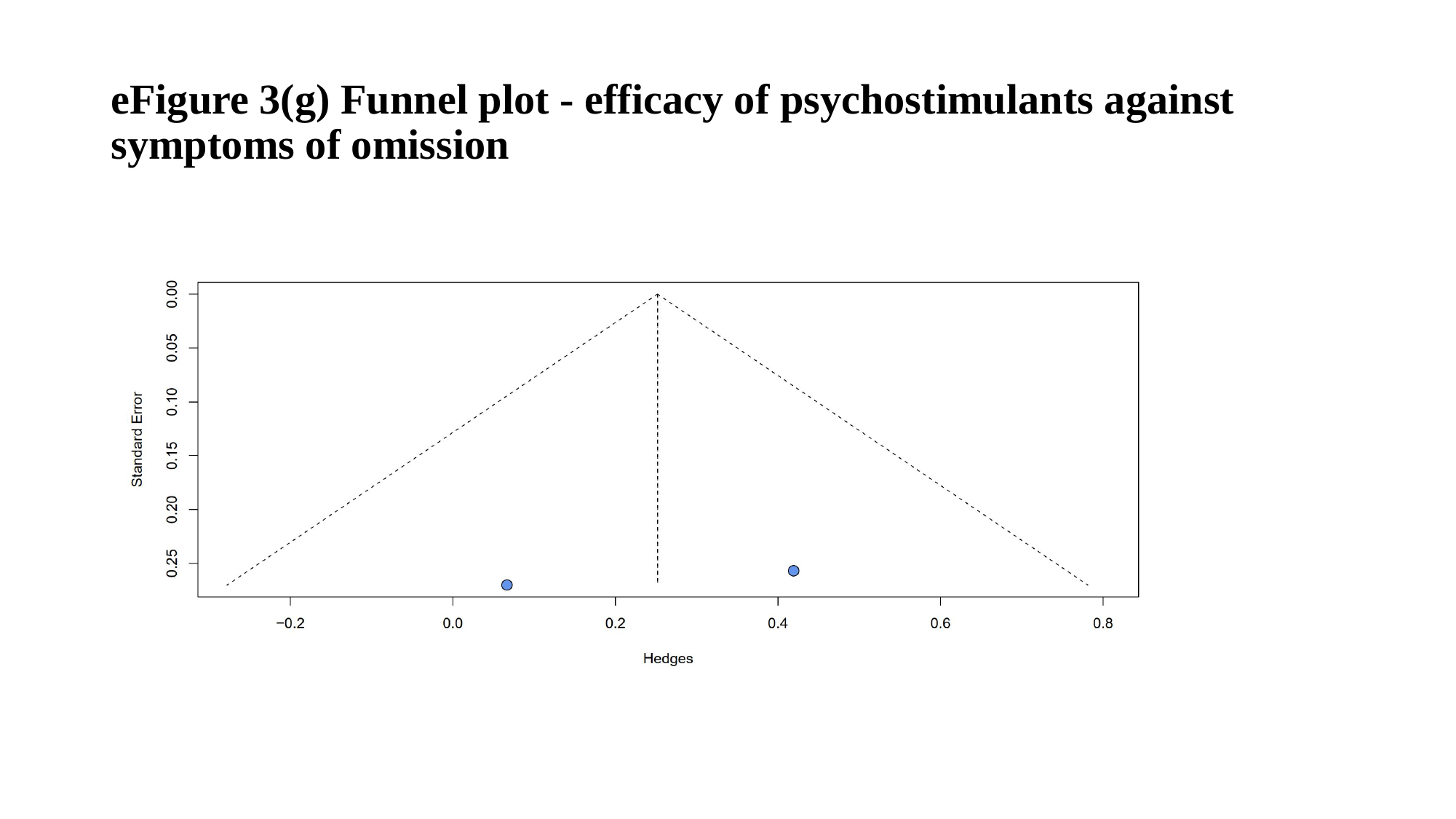

# eFigure 3(g) Funnel plot - efficacy of psychostimulants against symptoms of omission

## Slide 8
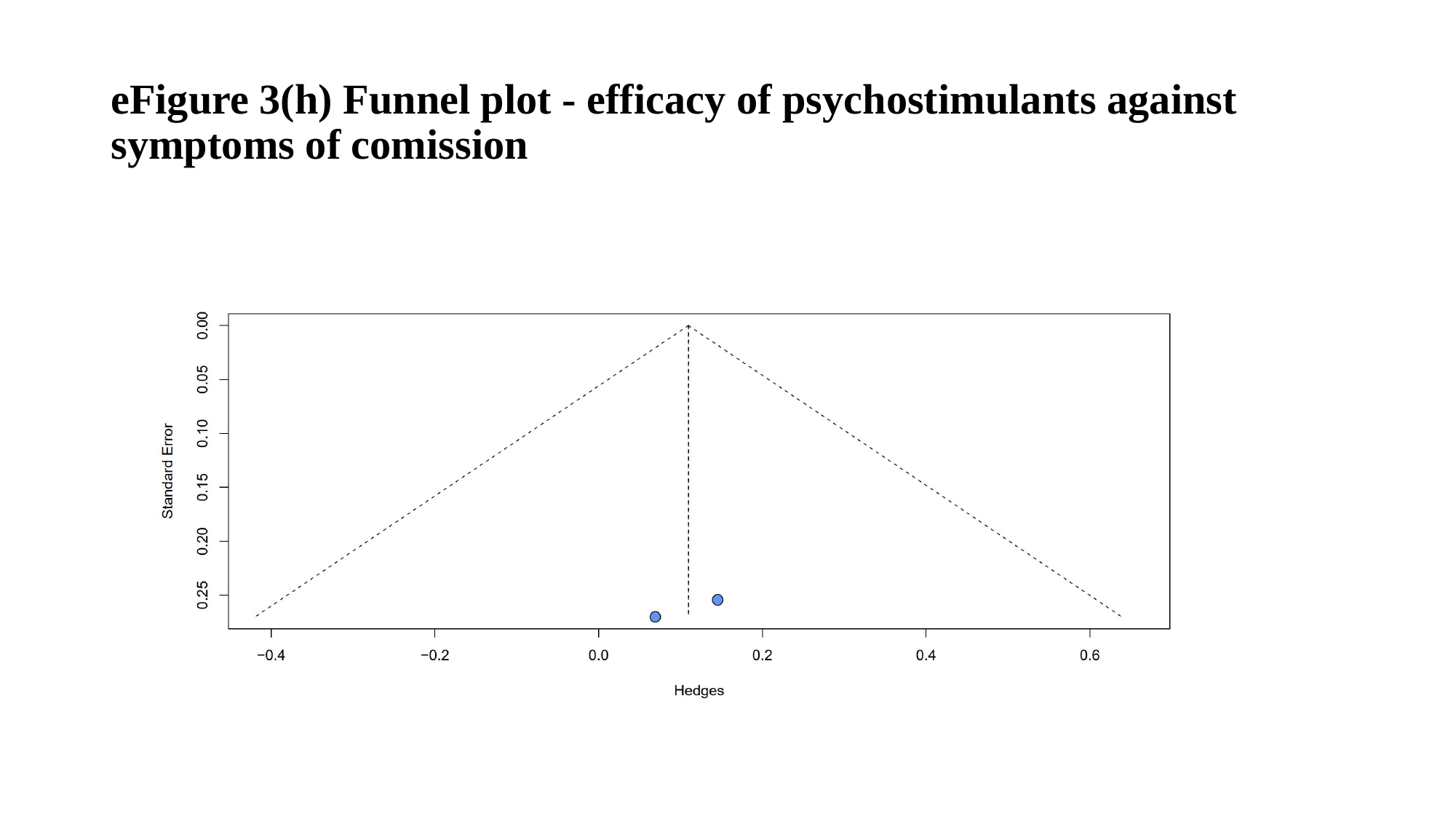

# eFigure 3(h) Funnel plot - efficacy of psychostimulants against symptoms of comission
