# Supplementary material for: Efficacy and tolerability of psychostimulants for symptoms of attention-deficit hyperactivity disorder in preschool children: A systematic review and meta-analysis
Source: Eur Psychiatry. 2023 Feb 15;66(1):e24. doi: 10.1192/j.eurpsy.2023.11 (PMC10044299; doi:10.1192/j.eurpsy.2023.11)
Supplement: Supplementary file 1 [file S0924933823000111sup001.zip › S0924933823000111sup004.docx]

**eTable 1. Applied keyword and the search result in each database**

| Database | Keywords | Filter | Date (yyyy/mm/dd) | Result |
| --- | --- | --- | --- | --- |
| PubMed | psychostimulants or methylphenidate or amphetamine | RCT  Preschool | 2022/04/28 | 98 |
| Embase | psychostimulants or methylphenidate or amphetamine | RCT  Preschool | 2022/04/28 | 150 |
| Cochrane CENTRAL | (psychostimulants or methylphenidate or amphetamine) AND preschool | Trials | 2022/04/28 | 240 |
| ScienceDirect | (psychostimulants or methylphenidate or amphetamine) AND preschool | Research article | 2022/04/28 | 65 |
| ClinicalTrials.gov | (ADHD) AND (psychostimulants) | NA | 2022/04/28 | 33 |

Abbreviations: NA, not applied; RCT, randomized controlled trial
