# Supplementary material for: Efficacy and tolerability of psychostimulants for symptoms of attention-deficit hyperactivity disorder in preschool children: A systematic review and meta-analysis
Source: Eur Psychiatry. 2023 Feb 15;66(1):e24. doi: 10.1192/j.eurpsy.2023.11 (PMC10044299; doi:10.1192/j.eurpsy.2023.11)
Supplement: Supplementary file 1 [file S0924933823000111sup001.zip › S0924933823000111sup005.docx]

**eTable 2. Reasons for study exclusion**

Not RCT (7)

[[1-7](#_ENREF_1)]

Included other age groups (10)

[[8-17](#_ENREF_8)]

Inadequate control group (3)

[[18-20](#_ENREF_18)]

Lack of adequate data for analysis (7)

[[21-27](#_ENREF_21)]

Duplicated sample source (4)

[[28-31](#_ENREF_28)]

Comparison groups not placebo (2)

[[32](#_ENREF_32), [33](#_ENREF_33)]

1. Childress, A.C., et al., *A Long-Term, Open-Label Safety and Tolerability Study of Lisdexamfetamine Dimesylate in Children Aged 4-5 Years with Attention-Deficit/Hyperactivity Disorder.* J Child Adolesc Psychopharmacol, 2022. **32**(2): p. 98-106.

2. Helseth, S.A. and D.A. Waschbusch, *Effects of behavioral and pharmacological therapies on peer reinforcement of deviancy in children with ADHD-only, ADHD and conduct problems, and controls.* 2015. **83**(2): p. 280-292.

3. Heriot, S.A., I.M. Evans, and T.M. Foster, *Critical influences affecting response to various treatments in young children with ADHD: a case series.* Child Care Health Dev, 2008. **34**(1): p. 121-33.

4. Nikles, C.J., et al., *Long-term changes in management following n-of-1 trials of stimulants in attention-deficit/hyperactivity disorder.* Eur J Clin Pharmacol, 2007. **63**(11): p. 985-9.

5. Charach, A., A. Ickowicz, and R. Schachar, *Stimulant treatment over five years: adherence, effectiveness, and adverse effects.* J Am Acad Child Adolesc Psychiatry, 2004. **43**(5): p. 559-67.

6. Findling, R.L., E.J. Short, and M.J. Manos, *Short-term cardiovascular effects of methylphenidate and adderall.* J Am Acad Child Adolesc Psychiatry, 2001. **40**(5): p. 525-9.

7. Corkum, P., P. Rimer, and R. Schachar, *Parental knowledge of attention-deficit hyperactivity disorder and opinions of treatment options: impact on enrollment and adherence to a 12-month treatment trial.* Can J Psychiatry, 1999. **44**(10): p. 1043-8.

8. Cutler, A.J., et al., *d-Amphetamine Transdermal System in Treatment of Children and Adolescents with ADHD: Secondary Endpoint Results from a Phase 2 Trial.* CNS Spectr, 2022. **27**(2): p. 230-231.

9. Chronis-Tuscano, A., et al., *Acute Effects of Parent Stimulant Medication Versus Behavioral Parent Training on Mothers' ADHD, Parenting Behavior, and At-Risk Children.* J Clin Psychiatry, 2020. **81**(5).

10. Froehlich, T.E., et al., *Sluggish Cognitive Tempo as a Possible Predictor of Methylphenidate Response in Children With ADHD: A Randomized Controlled Trial.* J Clin Psychiatry, 2018. **79**(2).

11. Mohammadpour, N., et al., *Effect of vitamin D supplementation as adjunctive therapy to methylphenidate on ADHD symptoms: A randomized, double blind, placebo-controlled trial.* Nutr Neurosci, 2018. **21**(3): p. 202-209.

12. Scahill, L., et al., *Using a Patient-Centered Outcome Measure to Test Methylphenidate Versus Placebo in Children with Autism Spectrum Disorder.* J Child Adolesc Psychopharmacol, 2017. **27**(2): p. 125-131.

13. Snircova, E., et al., *Anxiety reduction on atomoxetine and methylphenidate medication in children with ADHD.* Pediatr Int, 2016. **58**(6): p. 476-81.

14. Nikles, C.J., et al., *Aggregated n-of-1 trials of central nervous system stimulants versus placebo for paediatric traumatic brain injury--a pilot study.* Trials, 2014. **15**: p. 54.

15. Green, T., et al., *The effect of methylphenidate on prefrontal cognitive functioning, inattention, and hyperactivity in velocardiofacial syndrome.* J Child Adolesc Psychopharmacol, 2011. **21**(6): p. 589-95.

16. Findling, R.L., et al., *Methylphenidate in the treatment of children and adolescents with bipolar disorder and attention-deficit/hyperactivity disorder.* J Am Acad Child Adolesc Psychiatry, 2007. **46**(11): p. 1445-53.

17. Conklin, H.M., et al., *Acute neurocognitive response to methylphenidate among survivors of childhood cancer: a randomized, double-blind, cross-over trial.* J Pediatr Psychol, 2007. **32**(9): p. 1127-39.

18. Safavi, P., A.H. Dehkordi, and N. Ghasemi, *Comparison of the effects of methylphenidate and the combination of methylphenidate and risperidone in preschool children with attention-deficit hyperactivity disorder.* J Adv Pharm Technol Res, 2016. **7**(4): p. 144-148.

19. Pakdaman, F., et al., *The efficacy of Ritalin in ADHD children under neurofeedback training.* Neurol Sci, 2018. **39**(12): p. 2071-2078.

20. Akhondzadeh, S., M.R. Mohammadi, and M. Khademi, *Zinc sulfate as an adjunct to methylphenidate for the treatment of attention deficit hyperactivity disorder in children: a double blind and randomized trial [ISRCTN64132371].* BMC Psychiatry, 2004. **4**: p. 9.

21. Barkley, R.A., *The Effects of Methylphenidate on the Interactions of Preschool ADHD Children with Their Mothers.* Journal of the American Academy of Child & Adolescent Psychiatry, 1988. **27**(3): p. 336-341.

22. Waxmonsky, J.G., et al., *A Randomized Controlled Trial of Interventions for Growth Suppression in Children With Attention-Deficit/Hyperactivity Disorder Treated With Central Nervous System Stimulants.* J Am Acad Child Adolesc Psychiatry, 2020. **59**(12): p. 1330-1341.

23. Firestone, P., et al., *Short-term side effects of stimulant medication are increased in preschool children with attention-deficit/hyperactivity disorder: a double-blind placebo-controlled study.* J Child Adolesc Psychopharmacol, 1998. **8**(1): p. 13-25.

24. Kollins, S., et al., *Rationale, design, and methods of the Preschool ADHD Treatment Study (PATS).* J Am Acad Child Adolesc Psychiatry, 2006. **45**(11): p. 1275-1283.

25. Stein, M.A., et al., *Dopamine transporter genotype and methylphenidate dose response in children with ADHD.* Neuropsychopharmacology, 2005. **30**(7): p. 1374-82.

26. Aman, M.G., C. Binder, and A. Turgay, *Risperidone effects in the presence/absence of psychostimulant medicine in children with ADHD, other disruptive behavior disorders, and subaverage IQ.* J Child Adolesc Psychopharmacol, 2004. **14**(2): p. 243-54.

27. Efron, D., F.C. Jarman, and M.J. Barker, *Child and parent perceptions of stimulant medication treatment in attention deficit hyperactivity disorder.* J Paediatr Child Health, 1998. **34**(3): p. 288-92.

28. Abikoff, H.B., et al., *Methylphenidate effects on functional outcomes in the Preschoolers with Attention-Deficit/Hyperactivity Disorder Treatment Study (PATS).* J Child Adolesc Psychopharmacol, 2007. **17**(5): p. 581-92.

29. Mc, G.J., et al., *Pharmacogenetics of methylphenidate response in preschoolers with ADHD.* J Am Acad Child Adolesc Psychiatry, 2006. **45**(11): p. 1314-1322.

30. Wigal, T., et al., *Safety and tolerability of methylphenidate in preschool children with ADHD.* J Am Acad Child Adolesc Psychiatry, 2006. **45**(11): p. 1294-1303.

31. Swanson, J., et al., *Stimulant-related reductions of growth rates in the PATS.* J Am Acad Child Adolesc Psychiatry, 2006. **45**(11): p. 1304-1313.

32. Arabgol, F., L. Panaghi, and V. Nikzad, *Risperidone Versus Methylphenidate in Treatment of Preschool Children With Attention-Deficit Hyperactivity Disorder.* Iran J Pediatr, 2015. **25**(1): p. e265.

33. van der Veen-Mulders, L., et al., *Methylphenidate Has Superior Efficacy Over Parent-Child Interaction Therapy for Preschool Children with Disruptive Behaviors.* J Child Adolesc Psychopharmacol, 2018. **28**(1): p. 66-73.
