# Supplementary material for: Efficacy and tolerability of psychostimulants for symptoms of attention-deficit hyperactivity disorder in preschool children: A systematic review and meta-analysis
Source: Eur Psychiatry. 2023 Feb 15;66(1):e24. doi: 10.1192/j.eurpsy.2023.11 (PMC10044299; doi:10.1192/j.eurpsy.2023.11)
Supplement: Supplementary file 1 [file S0924933823000111sup001.zip › S0924933823000111sup006.docx]

**eTable 3a. The comparison between psychostimulants and placebo for side effect profiles in preschool children**

|  | **Number of studies** | **Effect sizes (95% confidence interval) (Odds ratio)** | **Effect size**  ***p* value** | **Heterogeneity *I*^2^ (%)** |
| --- | --- | --- | --- | --- |
| Decreased appetite | 6 | 2.3899 [1.0807; 5.2852] | 0.0314* | 27.9% |
| Irritability | 6 | 1.0630 [0.6401; 1.7653] | 0.8133 | 0.0% |
| Sleep disturbances | 4 | 1.3120 [0.7421; 2.3197] | 0.3503 | 0.0% |
| Prone to cry | 4 | 1.3594 [0.5113; 3.6145] | 0.5383 | 0.0% |
| Repetitive behaviors | 4 | 0.8812 [0.4791; 1.6207] | 0.6841 | 18.2% |
| Anxiety | 4 | 1.3181 [0.5267; 3.2987] | 0.5551 | 0.0% |

**eTable 3b. The comparison of Blood pressure and heart rate between psychostimulants and placebo in preschool children**

|  | **Number of studies** | **Patients/Controls** | **Effect sizes (95% confidence interval)** | **Effect size *p* value** | **Heterogeneity *I*^2^ (%)** |
| --- | --- | --- | --- | --- | --- |
| SBP | 2 | 38/37 | 0.0994 [-0.3553; 0.5542] | 0.6682 | 0.0% |
| DBP | 3 | 64/63 | 0.1691 [-0.1801; 0.5183] | 0.3427 | 0.0% |
| HR | 2 | 263/263 | -0.1230 [-0.5765; 0.3305] | 0.5949 | 0.0% |

SBP: systolic blood pressure, DBP: diastolic blood pressure, HR: heart rate
