# Supplementary material for: Efficacy and tolerability of psychostimulants for symptoms of attention-deficit hyperactivity disorder in preschool children: A systematic review and meta-analysis
Source: Eur Psychiatry. 2023 Feb 15;66(1):e24. doi: 10.1192/j.eurpsy.2023.11 (PMC10044299; doi:10.1192/j.eurpsy.2023.11)
Supplement: Supplementary file 1 [file S0924933823000111sup001.zip › S0924933823000111sup007.docx]

**eTable 4. Summary of side effects of the included studies**

| Study | Medications | Dosage | N | Duration (weeks) | Drop-out | Drop-out due to AE | Common side effects | Serious side effects |
| --- | --- | --- | --- | --- | --- | --- | --- | --- |
| Childress AC (2020) | MPH-MLR | Best dose (27.5mg/d) | 40 | 2 | N/A | 1 | hypertension, and emotional poverty, negativism, pollakiuria, onychophagia, decreased appetite, and tachycardia | Nil |
|  | Placebo |  | 50 |  | N/A | N/A |  |  |
| Ghuman JK (2009) | MPH | Best dose (14.46mg/d) | 14 | 4 | N/A | 1 | Upset Stomach, Increased Stereotypy, crying, difficulty falling asleep, restless sleep/waking up early | Nil |
|  | Placebo |  | 14 |  | N/A | N/A |  |  |
| Abikoff HB (2007) | MPH | Best dose | 61 | 4 | 10 | 1 | N/A | Nil |
|  | Placebo |  | 53 |  | 24 | 0 |  |  |
| Greenhill L (2006) | MPH | 3.75-22.5mg/d | 165 | 1 | N/A | 3 | appetite loss, trouble sleeping, stomachaches, social withdrawal, and lethargy | seizure (1) |
|  | Placebo |  | 165 |  | N/A | N/A |  |  |
| Chacko A (2004) | MPH | 0.6-1.2mg/kg/d | 36 | 6 | N/A | N/A | appetite loss, worry/anxiety, and tearfulness/depression | Nil |
|  | Placebo |  | 36 |  | N/A | N/A |  |  |
| Short EJ (2004) | MPH IR or MAS | Best dose | 28 | 3~4 | N/A | 0 | decreased appetite, irritability, crying, and rebound effects | Nil |
|  | Placebo |  | 28 |  | N/A | 0 |  |  |
| Handen BL (1999) | MPH | 0.6-1.2mg/kg/d | 11 | 3 | 1 | 1 | Poor appetite, Social withdrawal, Dull, not alert, Restless | Nil |
|  | Placebo |  | 11 |  | 0 | 0 |  |  |
| Musten LM (1997) | MPH | 0.6-1.0 mg/kg/d | 31 | 1 | N/A | N/A | N/A | Nil |
|  | Placebo |  | 31 |  | N/A | N/A |  |  |
| CK Conners (1975) | MPH | Best dose | 29 | 6 | N/A | N/A | insomnia, anorexia, ataxia, nausea, headache, vomiting, jitteriness, sadness | Nil |
|  | Placebo |  | 26 |  | N/A | N/A |  |  |

AE: adverse events; d: day; MPH: Methylphenidate; MPH-MLR: Methylphenidate extended-release, N/A Not available;
