# Supplementary material for: Efficacy and tolerability of psychostimulants for symptoms of attention-deficit hyperactivity disorder in preschool children: A systematic review and meta-analysis
Source: Eur Psychiatry. 2023 Feb 15;66(1):e24. doi: 10.1192/j.eurpsy.2023.11 (PMC10044299; doi:10.1192/j.eurpsy.2023.11)
Supplement: Supplementary file 1 [file S0924933823000111sup001.zip › S0924933823000111sup008.docx]

**eTable 5. Sensitivity analyses for meta-analysis using leave-one-out approach**

| Outcomes | Number of studies | Effect sizes  (95% confidence interval) | Effect size *p* value | Heterogeneity I^2^ (%) |
| --- | --- | --- | --- | --- |
| Parents inattention | 3 | 0.3410 (0.1446; 0.5374) | 0.0007 | 31.0% |
| **Leave one study out** |  |  |  |  |
| Greenhill L (2006) | 2 | 0.3631 [-0.3340; 1.0602] | 0.3074 | 62.7% |
| Teacher inattention | 3 | 0.2950 (0.1015; 0.4886) | 0.0028 | 0.0% |
| **Leave one study out** |  |  |  |  |
| Greenhill L (2006) | 2 | 0.3501 (-0.3058; 1.0060) | 0.2954 | 46.2% |
| Hyperactivity & Impulsivity(Teacher) | 3 | 0.5933 (0.0539; 1.1327) | 0.0311 | 63.4% |
| **Leave one study out** |  |  |  |  |
| Greenhill L (2006) | 2 | 0.8894 (-0.1280; 1.9069) | 0.0866 | 72.3% |
| Abikoff HB (2007) | 2 | 0.8149 (-0.3213; 1.9512) | 0.1598 | 81.5% |
