# Supplementary figures and images for: Efficacy and tolerability of psychostimulants for symptoms of attention-deficit hyperactivity disorder in preschool children: A systematic review and meta-analysis
Source: Eur Psychiatry. 2023 Feb 15;66(1):e24. doi: 10.1192/j.eurpsy.2023.11 (PMC10044299; doi:10.1192/j.eurpsy.2023.11)

## Slide 1
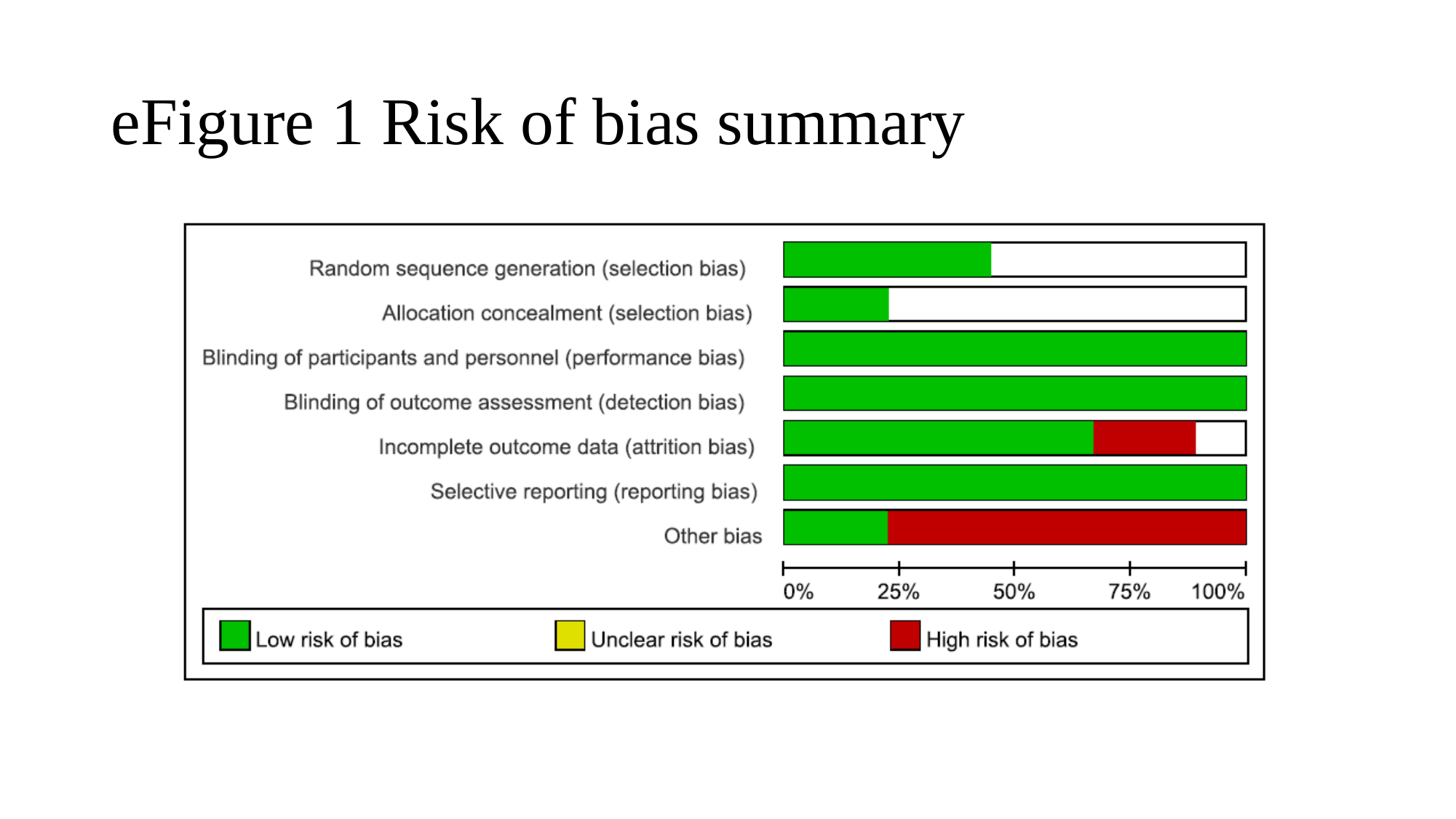

# eFigure 1 Risk of bias summary

Supplement: Supplementary file 1 [file S0924933823000111sup001.zip › S0924933823000111sup001.pptx]

## Slide 1
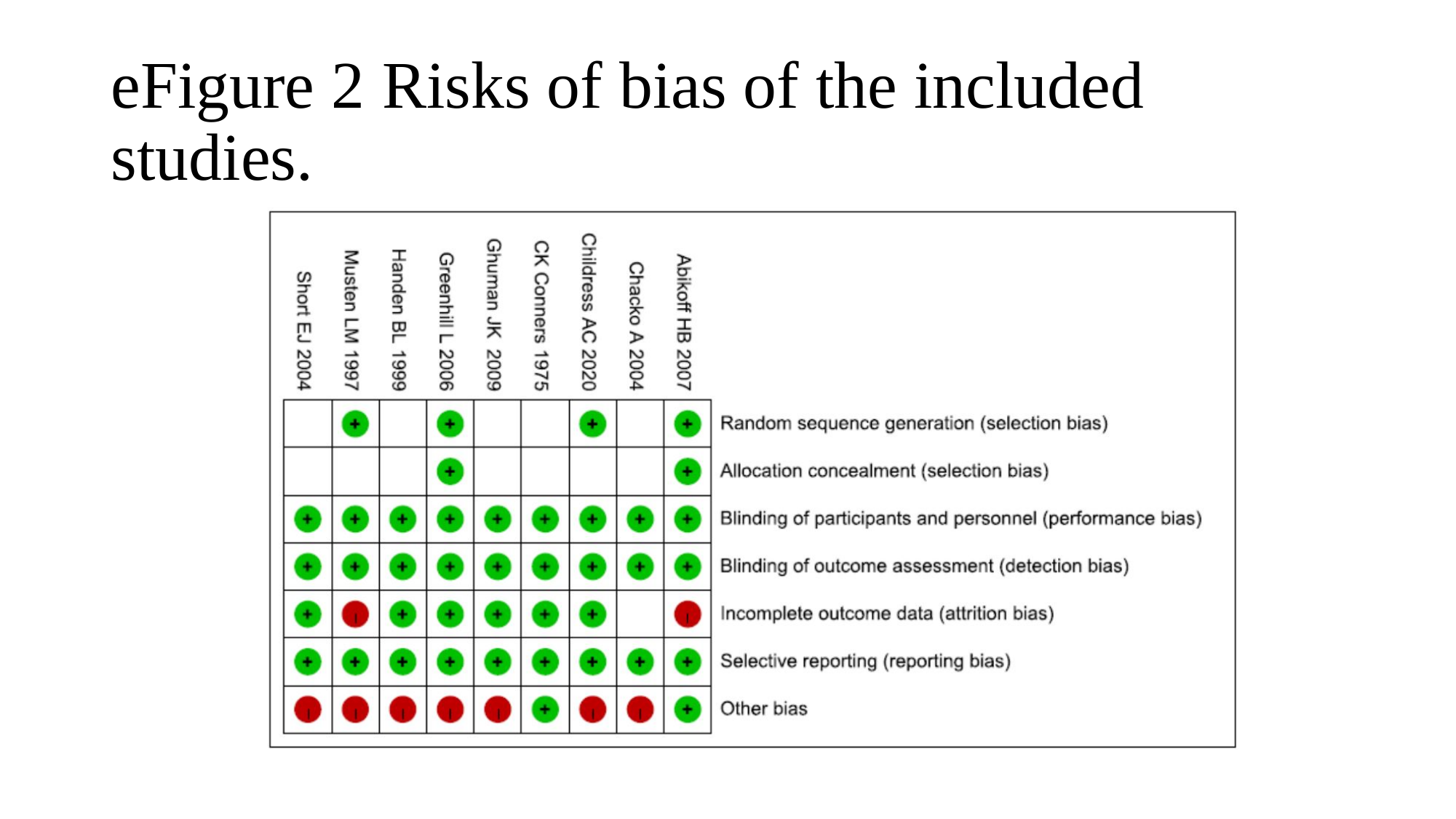

# eFigure 2 Risks of bias of the included studies.

Supplement: Supplementary file 1 [file S0924933823000111sup001.zip › S0924933823000111sup002.pptx]
