# Supplementary material for: Efficacy and tolerability of psychostimulants for symptoms of attention-deficit hyperactivity disorder in preschool children: A systematic review and meta-analysis
Source: Eur Psychiatry. 2023 Feb 15;66(1):e24. doi: 10.1192/j.eurpsy.2023.11 (PMC10044299; doi:10.1192/j.eurpsy.2023.11)
Supplement: Supplementary file 1 [file S0924933823000111sup001.zip › S0924933823000111sup009.doc]

**eTable 6.** Grading of Recommendations Assessments, Development and Evaluation (GRADE) assessment of the strength of evidence for standard weighted meta-analysis

| Outcome | Design | Risk of bias | Indirectness | Inconsistency | Imprecision | Publication bias | Grade Quality |
| --- | --- | --- | --- | --- | --- | --- | --- |
| Parent Total score | RCTx6 | No serious | No serious  indirectness | Serious  inconsistency | No serious  imprecision | Serious  publication bias | ⊕⊕ΟΟ^3,4^ |
| Teacher Total score | RCT x5 | No serious | No serious  indirectness | Serious  inconsistency | Serious  imprecision | Serious  publication bias | ⊕ΟΟΟ^3,4^ |
| Parents inattention | RCT x 3 | Serious | No serious  indirectness | Serious  inconsistency | Serious  imprecision | No serious  publication bias | ⊕ΟΟΟ^1,3,4^ |
| Teacher inattention | RCT x 3 | No serious | No serious  indirectness | Serious  inconsistency | Serious  imprecision | No serious publication bias | ⊕⊕ΟΟ^4,5^ |
| Hyperactivity & Impulsivity(Parents) | RCT x 4 | No serious | No serious  indirectness | Serious  inconsistency | Serious  imprecision | No serious  publication bias | ⊕⊕ΟΟ^3,4^ |
| Hyperactivity & Impulsivity(Teacher) | RCT x 3 | No serious | No serious  indirectness | Serious  inconsistency | Serious  imprecision | Serious publication bias | ⊕ΟΟΟ^3,4,5^ |
| Omission | RCT x 2 | Serious | No serious  indirectness | No serious  inconsistency | Serious  imprecision | Serious  publication bias | ⊕ΟΟΟ^1,4,5^ |
| Commission | RCT x 2 | Serious | No serious  indirectness | No serious  inconsistency | Serious  imprecision | Serious  publication bias | ⊕ΟΟΟ^1,4,5^ |

*Risk of bias was estimated using Cochrane risk of bias , studies were classified as having low risk of bias if none of the domains above was rated as high risk of bias and three or less were rated as unclear risk; moderate if one was rated as high risk of bias or none was rated as high risk of bias but four or

more were rated as unclear risk, and all other cases were assumed to pertain to high risk of bias.

Down-graded due to: ^1^ risk of bias, ^2^ indirectness, ^3.^ Inconsistency, ^4.^ Imprecision, ^5.^ publication bias
